# Usefulness of N-terminal pro-brain natriuretic peptide and C-reactive protein to predict ICU mortality in unselected medical ICU patients: a prospective, observational study

**DOI:** 10.1186/cc10004

**Published:** 2011-01-28

**Authors:** Feilong Wang, Wenzhi Pan, Shuming Pan, Shuyun Wang, Qinmin Ge, Junbo Ge

**Affiliations:** 1 Department of Emergency, Xinhua Hospital of Shanghai Jiaotong University, NO.1665, Kongjiang Road, Shanghai, 200092, PR China; 2 Department of Cardiology, Shanghai Institute of Cardiovascular Diseases, Zhongshan Hospital of Fudan University, NO.180, Fenglin Road, Shanghai, 200032, PR China

## Abstract

**Introduction:**

The performance of N-terminal pro-brain natriuretic peptide (NT-proBNP) and C-reactive protein (CRP) to predict clinical outcomes in ICU patients is unimpressive. We aimed to assess the prognostic value of NT-proBNP, CRP or the combination of both in unselected medical ICU patients.

**Methods:**

A total of 576 consecutive patients were screened for eligibility and followed up during the ICU stay. We collected each patient's baseline characteristics including the Acute Physiology and Chronic Health Evaluation II (APACHE-II) score, NT-proBNP and CRP levels. The primary outcome was ICU mortality. Potential predictors were analyzed for possible association with outcomes. We also evaluated the ability of NT-proBNP and CRP additive to APACHE-II score to predict ICU mortality by calculation of C-index, net reclassification improvement (NRI) and integrated discrimination improvement (IDI) indices.

**Results:**

Multiple regression revealed that CRP, NT-proBNP, APACHE-II score and fasting plasma glucose independently predicted ICU mortality (all *P *< 0.01). The C-index with respect to prediction of ICU mortality of APACHE II score (0.82 ± 0.02; *P *< 0.01) was greater than that of NT-proBNP (0.71 ± 0.03; *P *< 0.01) or CRP (0.65 ± 0.03; *P *< 0.01) (all *P *< 0.01). As compared with APACHE-II score (0.82 ± 0.02; *P *< 0.01), combination of CRP (0.83 ± 0.02; *P *< 0.01) or NT-proBNP (0.83 ± 0.02; *P *< 0.01) or both (0.84 ± 0.02; *P *< 0.01) with APACHE-II score did not significantly increase C-index for predicting ICU mortality (all *P *> 0.05). However, addition of NT-proBNP to APACHE-II score gave IDI of 6.6% (*P *= 0.003) and NRI of 16.6% (*P *= 0.007), addition of CRP to APACHE-II score provided IDI of 5.6% (*P *= 0.026) and NRI of 12.1% (*P *= 0.023), and addition of both markers to APACHE-II score yielded IDI of 7.5% (*P *= 0.002) and NRI of 17.9% *(P *= 0.002). In the cardiac subgroup (*N *= 213), NT-proBNP but not CRP independently predicted ICU mortality and addition of NT-proBNP to APACHE-II score obviously increased predictive ability (IDI = 10.2%, *P *= 0.018; NRI = 18.5%, *P *= 0.028). In the non-cardiac group (*N *= 363), CRP rather than NT-proBNP was an independent predictor of ICU mortality.

**Conclusions:**

In unselected medical ICU patients, NT-proBNP and CRP can serve as independent predictors of ICU mortality and addition of NT-proBNP or CRP or both to APACHE-II score significantly improves the ability to predict ICU mortality. NT-proBNP appears to be useful for predicting ICU outcomes in cardiac patients.

## Introduction

N-terminal pro-brain natriuretic peptide (NT-proBNP) is the inactive polypeptide of the pre-prohormone brain natriuretic peptide (BNP). It is synthesized in the cardiac myocytes in response to hemodynamic stress [1] or inflammatory status [2]. Over the last decade, some studies have indicated that NT-proBNP testing greatly increased the accuracy of the diagnosis of heart failure in patients with dyspnea [3,4]. NT-proBNP can also serve as a novel, independent predictor of prognosis in cardiovascular patients [5-7] as well as in the general population [8]. During the past few years, several studies [9-16] have focused on the potential value of NT-proBNP for prognosis of intensive care unit (ICU) patients, but the performance of NT-proBNP to predict adverse outcome in those patients is unimpressive [17]. First, the results of those studies have been conflicting. Several studies have shown that NT-proBNP could serve as an independent predictor of greater mortality in patients with cardiogenic shock [9], septic shock [10], severe sepsis [11], as well as in noncardiac [12-14] or unselected ICU patients [15], while another study [16] demonstrated that NT-proBNP failed to predict short-term mortality of ICU patients with hypoxic respiratory failure. Second, most of these studies were rather small and confounded by some factors, such as cardiovascular disease, renal insufficiency, or inflammation [17], although the prevalence of these conditions among patients admitted to ICU is generally high.

C-reactive protein (CRP) is an extremely sensitive objective marker of inflammation, tissue damage, and infection. Its ability to provide predictive value of long-term outcomes in ICU patients was just investigated in limited studies [18-20]. There were less data about the predictive value of CRP for short-term mortality [21,22]. In addition, although NT-proBNP and CRP have been shown to be predictors of adverse outcomes in ICU patients, the predictive value of the combination of both for outcomes has not been investigated.

Currently, the Acute Physiology and Chronic Health Evaluation II (APACHE-II) score is one of the most common models used to evaluate ICU patients' condition and predict their outcomes [23]. The additive ability of NT-proBNP and CRP to APACHE-II score to predict ICU mortality has rarely been assessed. Traditionally, predictive models have been evaluated by C-statistic, but this method has been criticized as being insensitive in comparing models [24] and for having little direct clinical relevance [25]. Several new methods have recently been proposed to evaluate and compare predictive risk models [26]. Calculation of net reclassification improvement (NRI) and integrated discrimination improvement (IDI) indices is now frequently being used [27]. We hypothesized that the higher plasma level of NT-proBNP and CRP would be independently associated with worse clinical outcomes in unselected ICU patients. We, therefore, undertook a prospective, observational study to assess the prognostic value of NT-proBNP, CRP or combination of both in a large population of unselected medical ICU patients. We also evaluated the ability of NT-proBNP and CRP additive to APACHE-II score to predict ICU mortality by calculation of C-index, NRI and IDI indices.

## Materials and methods

### Participants

The prospective, observational trial was undertaken between January 2009 and March 2010 at Xinhua Hospital Affiliated to Shanghai Jiaotong University School of Medicine. Medical patients were eligible for enrollment if they needed to be transferred to ICU from emergency department or other departments of our hospital (trauma and surgical patients were not included). The decision to transfer the patients into or out of ICU was made by at least one critical care expert and one medical expert. Exclusion criteria were age < 18 years and known pregnancy. Patients who died within four hours of admission or were discharged from the ICU within four hours of admission were also excluded because data collection for those patients was difficult. Patients were classified as cardiac or noncardiac subgroups according to their primary diagnosis. Noncardiac was defined as a patient with a primary noncardiac diagnosis. Noncardiac did not preclude a secondary cardiac disease, nor was a preexisting cardiac disease *a priori *excluded. The study was approved by Shanghai Jiaotong University Xinhua Hospital Ethics Committee (XHEC2011-002) and in accordance with the Declaration of Helsinki. Because this was an observational study and all laboratory indices (including CRP and NT-pro-BNP) observed were commonly measured for all patients in our ICU department, the need for written informed consent was waived by the review ethical review board.

### Laboratory methods

The NT-proBNP level was determined using the Elecsys Electro-chemo luminescent assay (Cobase 411 analyzer; Roche Diagnostics; Mannheim, Germany). The analytical range for NT-proBNP in the laboratory of our hospital is 5 to 35,000 pg/mL. Readings > 35,000 pg/mL were taken as 350,000 pg/mL. Reported total coefficient of variation is 4.4% at mean concentration 248.9 ng/L and 3.91% at MC 5,449 ng/L, respectively, based on multicenter calibrations of the automated Roche NT-proBNP assay [28]. Serum creatinine (SCr) and albumin were measured by the Hitachi 7600-120 (Hitachi, Tokyo, Japan) analyzer. We calculated the estimated glomerular filtration rate (eGFR) using the abbreviated Modification of Diet in Renal Disease (MDRD) study equation: eGFR (expressed in mL/minute/1.73 m2) = 186 * (SCr) -1.154 * (age) -0.203 *0.742 (if female), where SCr is serum creatinine in mg/dL [29]. Serum CRP levels were measured using Quick Read CRP test kit (Orion Corporation, Orion Diagnostica, Espoo, Finland). Blood samples were obtained from patients when they were admitted to ICU for measurement of the indicators mentioned previously.

### Study outcomes

At baseline, demographic and clinical characteristics, including the APACHE-II score (which can range from 0 to 71, with higher scores indicating more severe illness), were collected. Then the patients were followed up during the ICU stay. The primary outcome of this analysis was death in the ICU from any cause.

### Statistical analysis

Continuous variables and categorical variables were presented as mean value ± SD and %, respectively. But CRP, NT-proBNP and eGFR values were reported as median (95% confidence interval) and then logarithmically normalized (presented as log-CRP, log-NT-proBNP and log-eGFR, respectively) for statistical calculations because they were skewed. Baseline characteristics between survivals and non-survivals were compared with unpaired Student's *t*-test or Mann-Whitney test for continuous variables and chi-square or Fisher's exact tests for categorical variables. Univariate logistic regression analyses were performed to examine the association between mortality and each of the predictors separately. We also conducted a forward stepwise multivariate logistic regression to determine the independent predictors of ICU mortality. A criterion of *P *< 0.05 for entry and a *P *≥ 0.10 for removal was imposed in this procedure. Cox & Snell R Square and Nagelkerke R Square were calculated for assessing the goodness of fit of the models [30]. Odds ratios (ORs) for continuous variables were described using standardized ORs, which were associated with a one standard deviation change in the variable. The receiver operating characteristic (ROC) curve was used to examine the performance of variables to predict ICU mortality. The curve represented a plot of sensitivity vs 1-specificity. The area under the curve (AUC, that is, C-index) was calculated from the ROC curve. A statistically derived value, based on the Youden index, maximizing the sum of the sensitivity and specificity was used to define the optimal cut-off value [31]. ROC curve was also constructed for the combination of two or three variables for predicting ICU mortality according to the Mackinnon and Mulligan's weighted sum rule [32]. The differences between AUC (C-index) were tested by Hanley-McNeil methods in order to examine whether the addition of one or both of the biomarkers improved the discrimination of the model [33]. The increased discriminative value of the biomarkers was further examined by calculation of NRI and IDI indices described by Pencina *et al*. [27]. NRI is the net increase versus decrease in risk categories among case patients minus that among control participants. It requires that there exist *a priori *meaningful risk categories (we used < 10%, 10% to 30%, and 30% to 50%, and > 50% for the risk of ICU death) [26]. IDI is the difference in Yates slopes between models, in which the Yates slope is the mean difference in predicted probabilities between case patients and control participants [26]. A two-sided *P-*value of less than 0.05 was considered to indicate statistical significance. All analyses were performed with SPSS 13.0 software (SPSS Inc., Chicago, Illinois, USA).

## Results

### Baseline characteristics

In all, 576 consecutive patients (55.7% male; mean age 71.16 ± 16.5 years) were screened for eligibility. Baseline clinical and laboratory characteristics of the patients were shown in Table 1. For the full population, the median level of NT-proBNP, CRP and eGFR on admission was 2,922 (103 to 35,000.00) pg/ml, 39.8 (7.9 to 158.5) mg/L and 58.0 (6.5 to 150.0) mL/minute/1.73m^2^, respectively. The mean APACHE-Ⅱ score was 13.6 ± 7.1 points. The primary reasons for ICU admission were cardiovascular disease and pulmonary disease. A total of 41.9% of the patients had accompanying infections. A total of 131 (22.7%) patients died during the ICU hospitalization. Non-survivors were older and in a more severe condition as reflected by the higher APACHE-II score, were more frequently septic or infectious, had higher NT-proBNP, CRP, fasting plasma glucose, white blood cell and heart rate, and had lower eGFR and blood pressure on admission in the ICU as compared with survivors (Table 1).

**Table 1:** Baseline clinical and laboratory characteristics of subjects

	All	Survivors	Non-survivors	*P*-value
Patients, No.	576	445	131	/
Male (%)	55.7	57.8	58.0	1.000
Age (years)	71.5 ± 16.5	70.5 ± 16.0	74.9 ± 14.3	0.004
Principal diagnosis leading to ICU admission (%)				
Pulmonary disease	32.4	35.1	23.7	0.015
Cardiovascular disease	36.9	37.8	34.3	0.537
Neurologic disease	7.1	6.1	10.7	0.082
Digestive disease	5.6	6.1	3.8	0.391
Renal insufficiency	1.9	2.2	0.7	0.270
Poisoning	3.3	3.8	1.5	0.470
Infectious disease/sepsis	5.6	2.7	15.2	< 0.001
Other	7.1	6.3	9.9	0.175
Hypertension (%)	58.0	58.7	55.7	0.548
Diabetes mellitus (%)	25.9	24.7	29.8	0.257
Accompanying infection (%)	41.9	42.9	45.0	0.035
Systolic pressure (mmHg)	124.9 ± 22.4	127.4 ± 21.2	116.8 ± 24.7	< 0.001
Diastolic pressure (mmHg)	68.0 ± 12.2	69.3 ± 11.6	63.9 ± 13.3	< 0.001
Heart rate (bpm)	91.0 ± 20.5	89.2 ± 19.1	97.3 ± 24.0	< 0.001
Fasting plasma glucose (mmol/L)	11.4 ± 6.3	7.12 ± 2.9	8.5 ± 4.3	< 0.001
White blood cell (10^9^/L)	7.4 ± 3.4	10.8 ± 5.6	13.5 ± 7.9	< 0.001
Hemoglobin (g/L)	116.3 ± 25.4	118.7 ± 24.0	108.1 ± 28.1	< 0.001
eGFR(mL/min/1.73 m^2^)	58.0 (6.5 to 150.0)	62.6 (14.5 to 30.2)	34.8 (8.2 to 108.7)	< 0.001
APACHE-II score (points)	13.6 ± 7.1	11.7 ± 5.6	19.9 ± 7.5	< 0.001
NT-proBNP (ng/ml)	2,922 (103 to 35,000)	5,996 (6 to 35,000)	12,726 (59 to 35,000)	< 0.001
CRP(mg/L)	39.8 (7.9 to 158.5)	56.7 (1.0 to 160.1)	85.9 (6.0 to 160.2)	< 0.001

APACHE II score, Acute Physiology and Chronic Health Evaluation II score; CRP, C-reactive protein; eGFR, estimated glomerular filtration rate; NT-proBNP, N-terminal pro-brain natriuretic peptide.

### Predictors of ICU mortality

Univariate logistic regression analysis demonstrated that those older, with higher level of NT-proBNP, CRP and fasting plasma glucose, higher APACHE-II score and lower eGFR had significantly greater hazard of death (Table 2) (Because blood pressure, heart rate, white blood cell counts and hemoglobin levels had been included in APACHE-II score, they did not enter into the analysis). When all the observed baseline variables (Table 2) were included in a stepwise multiple logistic model in which ICU mortality was the dependent variable; only CRP, log-NT-proBNP, APACHE-II score and fasting plasma glucose could independently predict primary outcome (*P *= 0.032, 0.011, 0.000 and 0.039, receptively).

**Table 2:** Univariate odds ratios of variables for predicting ICU mortality

Predictor	Odds ratio	95% CI	*P*
Age	1.430	1.154 to 1.772	0.001
Sex	/	/	0.992
Log-NT-proBNP	2.202	1.728 to 2.807	< 0.001
Fasting plasma glucose	1.445	1.201 to 1.741	< 0.001
APACHE-II score	4.359	3.301 to 5.756	< 0.001
Log-eGFR	0.362	0.276 to 0.476	< 0.001
Log-CRP	1.768	1.439 to 2.173	< 0.001

Odds ratios for continuous variables shown as standardized odds ratios (OR per 1 SD). Log-variable is the logarithm of the variable. APACHE II score, Acute Physiology and Chronic Health Evaluation II score; CRP, C-reactive protein; NT-proBNP, eGFR, estimated glomerular filtration rate; N-terminal pro-brain natriuretic peptide.

### Value for CRP and NT-proBNP in prediction of ICU mortality

**Figure 1:**
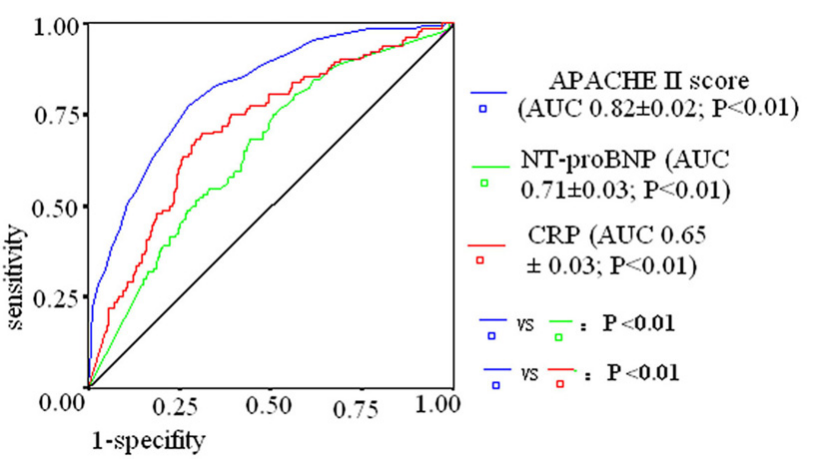
**ROC curves for APACHE II score, CRP and NT-proBNP in prediction of ICU mortality**. The area under the ROC curve (AUC) of NT-proBNP were larger than that of CRP or NT-proBNP (all *P *< 0.01).

To evaluate the value for the above independent variables to predict ICU mortality, ROC curves were drawn (Figure 1). The AUC was calculated as 0.82 ± 0.02 (*P *< 0.01) for APACHE II score, 0.71 ± 0.03 (*P *< 0.01) for NT-proBNP and 0.65 ± 0.03 (*P *< 0.01) for CRP. The AUC of NT-proBNP or CRP was lower than that of APACHE II score (all *P *< 0.01). The optimal cutoff value of APACHE II score for predicting death was ≥ 15, which gave sensitivity of 77.3% and specificity of 72.5%, and of NT-proBNP was ≥ 4,750 ng/ml, which provided sensitivity of 69.5% and specificity of 68.8%. The optimal cutoff value of CRP (≥ 27 mg/L) provided sensitivity of 75.05% and specificity of 49.5%.

### Combination of CRP or NT-proBNP or both with APACHE II score for predicting ICU mortality

**Figure 2:**
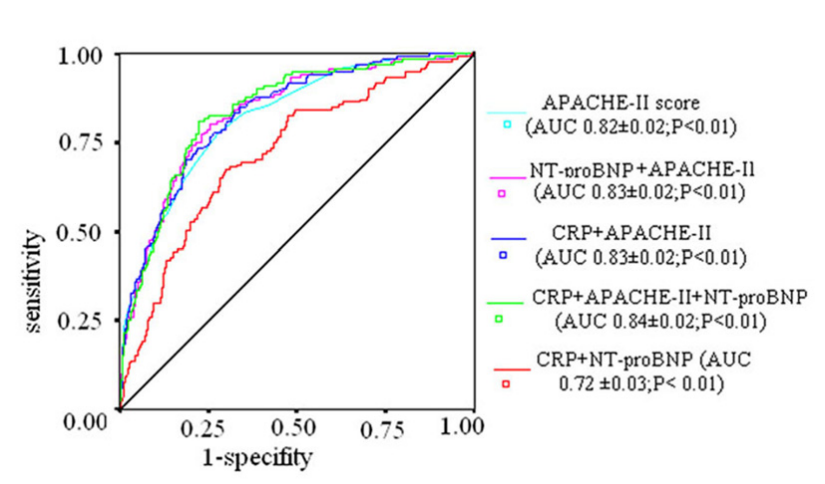
**ROC curves for combination of two or three variables among CRP, NT-proBNP and APACHE-II score**. Combination of CRP or NT-proBNP or both with APACHE-II score did not significantly increase AUC with regard to perdition of ICU mortality (all *P *> 0.05). Combination of CRP and NT-proBNP was inferior to APACHE-II score alone for predicting ICU mortality (*P *< 0.01).

To further clarify whether CRP or NT-proBNP or the combination of both had an additive power with APACHE-II score for predicting ICU mortality, we combined one or two biomarkers with the APACHE-II score to construct new ROC curves (Figure 2). As compared with the APACHE-II score (AUC 0.82 ± 0.02), combination of CRP (AUC 0.83 ± 0.02) or NT-proBNP (AUC 0.83 ± 0.02) or both (AUC 0.84 ± 0.02) with the APACHE-II score did not significantly increase AUC for predicting ICU mortality (*P *= 0.74, 0.74 and 0.47, respectively). The combination of CRP and NT-proBNP (AUC 0.72 ± 0.03) was inferior to APACHE-II score for predicting ICU mortality (*P *< 0.01). In addition, the forward stepwise logistic regression showed that the addition of NT-proBNP or both biomarkers to the APACHE-II score slightly increased the ability of the model to predict ICU mortality. The Cox & Snell R Square and Nagelkerke R Square in the model were slightly increased. (Table 3) However, when using new statistical analysis methods (NRI and IDI indices) which are more sensitive than the above statistics, we found that the addition of NT-proBNP or CRP or both to the APACHE-II score significantly improved the ability to predict the outcome (Table 4). The addition of NT-proBNP to he APACHE-II score gave an IDI of 6.6% (*P *= 0.003) and NRI of 16.6% (*P *= 0.007). The addition of CRP to the APACHE-II score provided an IDI of 5.6% (*P *= 0.026) and NRI of 12.1% (*P *= 0.023), and the addition of both markers to the APACHE-II score yielded an IDI of 7.5% (*P *= 0.002) and NRI of 17.9% *(P *= 0.002).

**Table 3:** NRI and IDI for assessing improvement in model performance after adding biomarkers to APACHE-II score

	NRI	Z value for NRI	*P-*value for NRI	IDI	Z value for NRI	*P-*value for NRI
APACHE-II score + log(NT-proBNP)	16.6%	2.982	0.003	6.6%	2.680	0.007
APACHE-II score + log(CRP)	12.1%	2.216	0.026	5.6%	2.267	0.023
APACHE-II score + log(NT-proBNP) +log(CRP)	17.9%	3.042	0.002	7.5%	3.036	0.002

APACHE II score, Acute Physiology and Chronic Health Evaluation II score; CRP, C-reactive protein; eGFR, estimated glomerular filtration rate; IDI, integrated discrimination improvement; NRI, Net reclassification improvement; N-terminal pro-brain natriuretic peptide; NT-proBNP

**Table 4:** Independent predictors of ICU mortality by multivariate logistic regression in all patients (appending models summary)

		OR	OR-st	*P*	-2 Log likelihood	Cox & Snell R Square	Nagelkerke R Square
Model I	APACHE-II score	1.225	4.359	0.000	467.269	0.215	0.328
Model II	Log-NT-proBNP	1.633	1.454	0.008	458.360	0.228	0.346
	APACHE-II score	1.196	3.532	0.000			
Model III	Log-CRP	1.778	1.355	0.017			
	Log-NT-proBNP	1.628	1.448	0.011	452.586	0.236	0.358
	APACHE-II score	1.183	3.225	0.000			

APACHE II score, Acute Physiology and Chronic Health Evaluation II score; CRP, C-reactive protein; eGFR, estimated glomerular filtration rate; OR, odds ratio; OR-st, standardized Odds ratios (OR per 1 SD); NT-proBNP, N-terminal pro-brain natriuretic peptide.

### Subgroups analysis

In the non-cardiac subgroup (*N *= 363), the AUC with respect to the prediction of ICU mortality was 0.82 ± 0.03 (*P *< 0.01) for the APACHE II score, 0.70 ± 0.03 (*P *< 0.01) for NT-proBNP and 0.64 ± 0.04 (*P *< 0.01) for CRP. The AUC of the APACHE II score was larger than that of NT-proBNP or CRP (*P *< 0.01). Multiple logistic regression showed that CRP and APACHE-II scores were independent predictors of ICU mortality (all *P *< 0.01; Table 5). However, the addition of CRP to the APACHE-II score in the model just slightly increased Cox & Snell R Square and Nagelkerke R Square (Table 5), and did not improve the AUC (0. 82 ± 0.03 vs 0.83 ± 0.03, *P *= 0.82). The IDI (2.89%, Z = 0.91, *P *= 0.33) and NRI (6.36%, Z = 1.15, *P *= 0.25) were also not statistically significant. In the cardiac subgroup (*N *= 213), the AUC with respect to the prediction of ICU mortality was calculated as 0.81 ± 0.03 (*P *< 0.01) for the APACHE II score, 0.77 ± 0.04 (*P *< 0.01) for NT-proBNP and 0.69 ± 0.04 (*P *< 0.01) for CRP. The AUC of the APACHE-II score was not different from that of NT-proBNP (*P *= 0.42), while the AUC of both was lager than that of CRP (*P *< 0.01). Multiple logistic regression analysis demonstrated that NT-proBNP and the APACHE-II score were independent predictors of ICU mortality (all *P *< 0.01; Table 6). The addition of NT-proBNP to the APACHE-II score can obviously increase Cox & Snell R Square and Nagelkerke R Square (Table 6). Although the AUC increased (0.82 ± 0.03 vs 0.86 ± 0.03, *P *= 0.35) insignificantly, the IDI (10.2%, Z = 2.55, *P *= 0.018) and NRI (18.5%, Z = 2.20, *P *= 0.028) were statistically significant.

**Table 5:** Independent predictors of ICU mortality by multivariate logistic regression in non-cardiac patients (appending models summary)

		OR	OR-st	*P*	-2 Log likelihood	Cox & Snell R Square	Nagelkerke R Square
Model I	APACHE-II score	1.207	3.987	0.000	295.870	0.230	0.346
Model II	Log-CRP	2.056	1.451	0.002	290.175	0.242	0.365
	APACHE-II score	1.198	3.781	0.000			

OR, odds ratio; OR-st, standardized Odds ratios (OR per 1 SD); APACHE II score, Acute Physiology and Chronic Health Evaluation II score; eGFR, estimated glomerular filtration rate; CRP, C-reactive protein; NT-proBNP, N-terminal pro-brain natriuretic peptide.

**Table 6:** Independent predictors of ICU mortality by multivariate logistic regression in cardiac patients (appending models summary)

		OR	OR-st	*P*	-2 Log likelihood	Cox & Snell R Square	Nagelkerke R Square
Model I	APACHE-II score	1.265	4.371	0.000	167.854	0.201	0.311
Model II	Log-NT-proBNP	3.356	2.296	0.002	157.161	0.241	0.373
	APACHE-II score	1.226	3.618	0.000			

APACHE II score, Acute Physiology and Chronic Health Evaluation II score; CRP, C-reactive protein; eGFR, estimated glomerular filtration rate; NT-proBNP, N-terminal pro-brain natriuretic peptide; OR, odds ratio; OR-st, standardized Odds ratios (OR per 1 SD).

## Discussion

In this large scale study of 576 unselected medical ICU patients, we found that NT-proBNP and CRP independently predicted ICU mortality even after adjustment for the APACHE II score and multiple potential confounders including eGFR, age, and so on. Although the predictive ability was lower as compared with the APACHE II score, the addition of CRP or NT-proBNP or both to the APACHE II score could significantly improve the ability to predict ICU mortality, as demonstrated by IDI and NRI indices. NT-proBNP appeared to be more useful for predicting ICU outcomes in cardiac patients. To our knowledge, this is the first large-scale study to evaluate the ability of NT-proBNP and CRP added to the APACHE-II score to predict ICU mortality, especially using the new statistics method, that is, the NRI and IDI indices.

BNP and NT-proBNP have become promising biomarkers recently. They have been used as tools for risk stratification in cardiac patients [3-7], the general population [8] and ICU patients [9-18]. Most of the studies investigating the predictive value of NT-proBNP in ICU patients were confounded by some factors, such as renal insufficiency or inflammation. Our study showed that NT-proBNP independently predicted ICU mortality in unselected patients even after adjustment for the APACHE II score and other potential confounders, including age, renal insufficiency (eGFR), and inflammation (CRP). However, the ability of NT-proBNP to predict ICU mortality was lower than that of the APACHE II score (AUC: 0.82 ± 0.02 vs 0.71 ± 0.03, *P *< 0.01; OR: 1.454 vs 3.532). The C statistic is the most commonly used method of determining model discrimination. In this method, we found that the addition of one or both the biomarkers to the APACHE II score did not significantly improve the predictive ability (AUC). However, the sole reliance on the C-statistic for the evaluation of predictors has been questioned, because very large independent associations of a new marker with the outcome are required to result in a significant increase in the C statistic [24,25]. In the present study, we also used a more sensitive test of improvement in model discrimination [27]. We found that the addition of NT-proBNP to the APACHE II score significantly increased the ability to predict ICU mortality as demonstrated by the IDI (6.6%, *P *= 0.003) and NRI (16.6%, *P *= 0.007) indices. NT-proBNP was not an independent predictor of ICU mortality in the non-cardiac subgroup after adjustment for APACHE II score and CRP. Kotanidou *et al*. [13] found that NT-proBNP predicted mortality independently after the adjusted APACHE II score and some inflammatory cytokines levels in non-cardiac ICU patients. But they used TNF-α, IL-6, and IL-10 rather than CRP and enrolled many surgical and multiple trauma cases. In the cardiac subgroup, NT-proBNP independently predicted ICU mortality while the AUC of the APACHE II score was not different from that of NT-proBNP (0.81 ± 0.03 vs 0.77 ± 0.04; *P *> 0.05). The addition of NT-proBNP to the APACHE-II score can obviously increase predictive ability (IDI = 10.2%, *P *= 0.018; NRI = 18.5%, *P *= 0.028). Therefore, although NT-proBNP could predict ICU mortality in unselected medical patents, it appeared to be more useful in cardiac patients than in non-cardiac patients.

LV wall tension is regarded as the primary mechanism regulating NT-proBNP secretion [1]. Other hemodynamic factors that may contribute to NT-proBNP secretion include left ventricular diastolic dysfunction and right ventricular overload and dysfunction [10,34]. Other mechanisms proposed to account for high NT-proBNP values include renal dysfunction [35] and inflammatory status [36,37]. Therefore, patients with high NT-proBNP may have cardiac dysfunction, renal dysfunction or inflammatory status. All of these factors have shown to be a frequent and important factors in determining the outcome of critically ill patients [18-21,38]. This is the reason why NT-proBNP can be used as a predictor of outcomes in ICU patients.

CRP has long been considered to be a distinct and sensitive biomarker of inflammation, tissue damage, and infection. Some studies also suggest that CRP may be an indicator of organ failure [22]. Only a few studies have tested its value for predicting outcome in ICU patients [18-22]. However, most of these studies observed the post-ICU outcomes but not ICU mortality. NT-proBNP was not included in their analyses, either. One previous study showed no predictive value of CRP for in-hospital mortality, even in univariate analysis [21]. The scope of the study was rather small (*N *= 103) and, thus, the statistical power was less than that of our study. Moreover, the endpoint of the previous study was in-hospital mortality but not ICU mortality. The present study revealed that CRP was also an independent predictor of ICU mortality in unselected patients or non-cardiac patients. Although the C-statistic showed the addition of CRP to the APACHE-II score in prediction of ICU mortality did not significantly improve the predictive ability, NRI (12.1%, *P *= 0.026) and IDI (5.6%, *P *= 0.023) were statistically significant.

Several limitations of our study should be mentioned. First, neither echocardiography was performed nor cardiac function assessed in the present study. The division of subgroups was according to primary admission cause. Thus patients in the non-cardiac group may also have cardiac disease and cardiac dysfunction. However, patients with cardiac diseases as the primary principal diagnosis leading to ICU admission must have cardiac diseases. The statistical conclusion drawn from the cardiac group was appropriate. Second, this was a single-center study, and participants did not include surgery and trauma patients. The value for NT-proBNP in prediction of adverse outcome would be a bit different if the population was different. At last, a limitation of the net reclassification improvement and other reclassification measures is that they depend on the particular categories used [26]. We had used < 10%, 10% to 30%, and 30% to 50%, and > 50% for the risk of ICU death as risk categories. But there are still no well-recognized risk categories now. If the risk categories used had been different, the NRI would be a bit different.

## Conclusions

In this large-scale study of unselected ICU patients, we confirmed that NT-proBNP and CRP can serve as moderate independent predictors of ICU mortality. Although the predictive ability was lower compared with the APACHE II score, but the addition of CRP or NT-proBNP or both to the APACHE II score could significantly improve the ability to predict ICU mortality, as demonstrated by IDI and NRI indices. NT-proBNP appeared to be more useful for predicting ICU outcomes in cardiac patients.

## Key messages

● NT-proBNP and CRP independently predicted ICU mortality even after adjustment for the APACHE II score and multiple potential confounders.

● The ability of NT-proBNP and CRP to predict ICU mortality was lower compared with the APACHE II score.

● The addition of CRP or NT-proBNP or both to the APACHE II score could significantly improve the ability to predict ICU mortality as demonstrated by IDI and NRI indices.

● NT-proBNP appeared to be more useful for predicting ICU outcomes in cardiac patients.

## Abbreviations

AUC: area under the curve; APACHE-II: score, Acute Physiology and Chronic Health Evaluation II score; BNP: brain natriuretic peptide; CRP.: -reactive protein; eGFR,: estimated glomerular filtration rate; ICU: intensive care unit; IDI: integrated discrimination improvement; MCV: mean corpuscular volume; MDRD: Modification of Diet in Renal Disease; NRI: net reclassification improvement; OR: odds ratio; RDW: red blood cell distribution width; ROC: curve, receiver operating characteristic curve; SCr: serum creatinine.

## Competing interests

The authors declare that they have no competing interests.

## Authors' contributions

WeP and JG participated in the design of the study and performed the statistical analysis and drafted the manuscript. FW and SP carried out data collection, contributed to the design of the study and helped to draft the manuscript. QG and SW participated in the data collection. All authors read and approved the final manuscript.
